# Circulating primitive erythroblasts establish a functional, protein 4.1R-dependent cytoskeletal network prior to enucleating

**DOI:** 10.1038/s41598-017-05498-4

**Published:** 2017-07-12

**Authors:** Yu-Shan Huang, Luis F. Delgadillo, Kathryn H. Cyr, Paul D. Kingsley, Xiuli An, Kathleen E. McGrath, Narla Mohandas, John G. Conboy, Richard E. Waugh, Jiandi Wan, James Palis

**Affiliations:** 1 Department of Biomedical Genetics, University of Rochester, Rochester, NY 14642 USA; 2 Department of Biomedical Engineering, University of Rochester, Rochester, NY 14642 USA; 3 Department of Biomedical Engineering, Rochester Institute of Technology, Rochester, NY 14623 USA; 4 Department of Pediatrics and Center for Pediatric Biomedical Research, University of Rochester, Rochester, NY 14642 USA; 5 Red Cell Physiology Laboratory, New York Blood Center, New York, NY 10065 USA; 6 Life Sciences Division, Lawrence Berkeley National Laboratory, Berkeley, CA 94720 USA; 7 Microsystems Engineering, Rochester Institute of Technology, Rochester, NY 14623 USA

## Abstract

Hematopoietic ontogeny is characterized by distinct primitive and definitive erythroid lineages. Definitive erythroblasts mature and enucleate extravascularly and form a unique membrane skeleton, composed of spectrin, 4.1R-complex, and ankyrinR-complex components, to survive the vicissitudes of the adult circulation. However, little is known about the formation and composition of the membrane skeleton in primitive erythroblasts, which progressively mature while circulating in the embryonic bloodstream. We found that primary primitive erythroblasts express the major membrane skeleton genes present in similarly staged definitive erythroblasts, suggesting that the composition and formation of this membrane network is conserved in maturing primitive and definitive erythroblasts despite their respective intravascular and extravascular locations. Membrane deformability and stability of primitive erythroblasts, assayed by microfluidic studies and fluorescence imaged microdeformation, respectively, significantly increase prior to enucleation. These functional changes coincide with protein 4.1 R isoform switching and protein 4.1R-null primitive erythroblasts fail to establish normal membrane stability and deformability. We conclude that maturing primitive erythroblasts initially navigate the embryonic vasculature prior to establishing a deformable cytoskeleton, which is ultimately formed prior to enucleation. Formation of an erythroid-specific, protein 4.1R-dependent membrane skeleton is an important feature not only of definitive, but also of primitive, erythropoiesis in mammals.

## Introduction

In the adult, definitive red blood cells require a functional erythroid-specific membrane skeletal network to maintain cell integrity while repeatedly deforming in the microcirculation. Since components of this network were first discovered over 40 years ago, considerable research has revealed that the erythroid-specific membrane skeleton consists of α- and β-spectrin connected through ankyrinR- and junctional protein 4.1R-complexes to the lipid bilayer^1, 2^. The anion exchanger band 3 is the most abundant protein in the red cell membrane skeleton and a component of both complexes^1, 2^. Another functionally important component of this network in adult mammalian red blood cells is the erythroid-specific isoform of protein 4.1R, generated through a complex process of lineage-specific splicing^3^. Defects of cytoskeletal proteins, including protein 4.1R, lead to morphologically abnormal, mechanically fragile erythrocytes, resulting in hemolytic anemias, including hereditary spherocytosis and hereditary elliptocytosis^4^.

**Figure 1:**
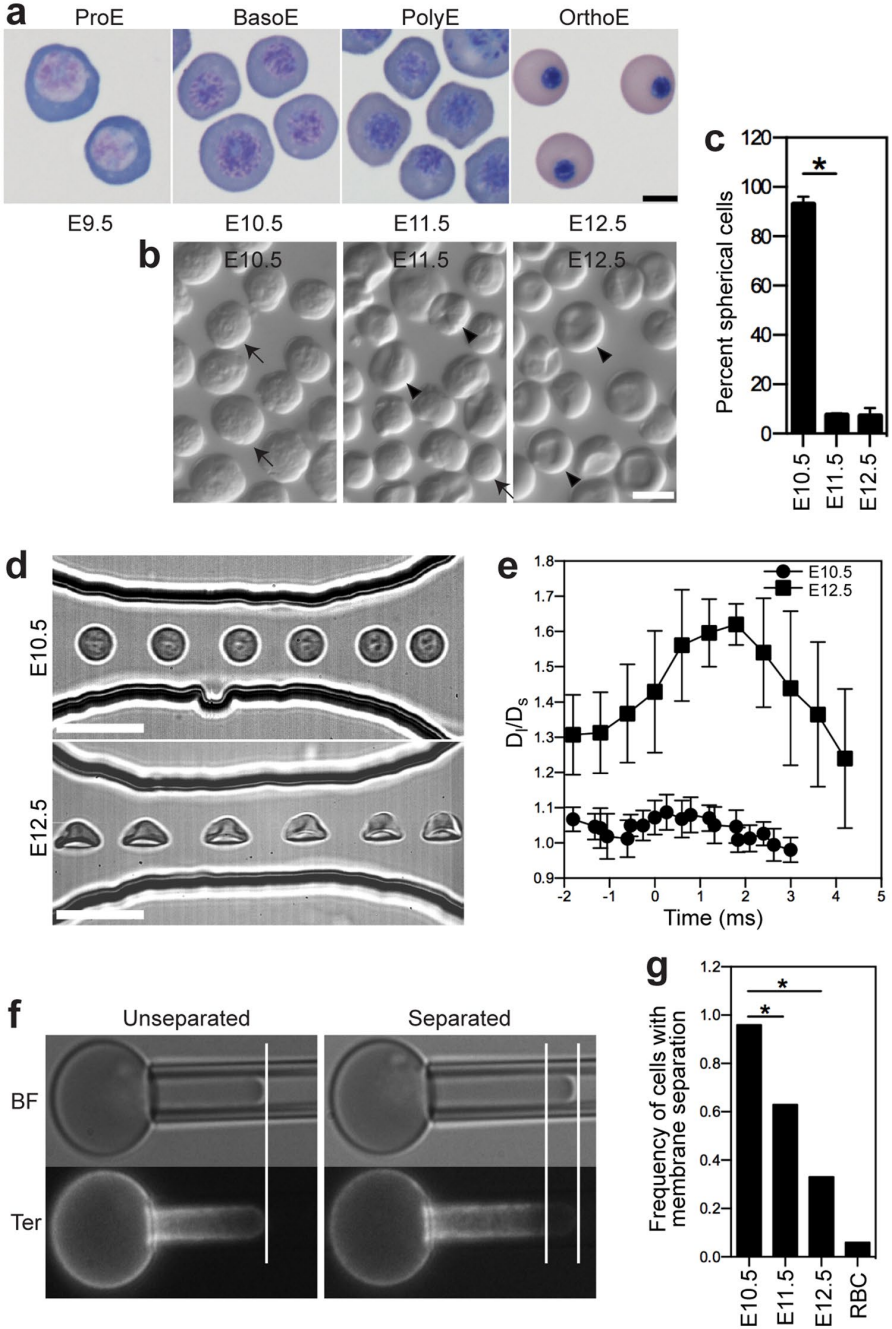
Primary primitive erythroblasts alter their cell shape, gain the capability to deform, and develop increasing mechanical stability as they transition from BasoE at E10.5 to OrthoE at E12.5. (**a**) Circulating primitive erythroblasts transition from ProE at E9.5 to OrthoE at E12.5 as a single cohort in the bloodstream. ProE, proerythroblasts; BasoE, basophilic erythroblasts; PolyE, polychromatophilic erythroblasts; PolyE/OrthoE, polychromatophilic and orthochromatic erythroblasts; and OrthoE, orthochromatic erythroblasts. (**b**) Primitive erythroblasts alter their cell shape from spherical to partially concave as they progress from BasoE at E10.5 to OrthoE at E12.5. Freshly isolated fetal blood cells were visualized by light microscopy. Cells were identified as being spherical (arrows) versus partially concave (arrow heads). Scale bar represents 10 µm. (**c**) The proportion of spherical primitive erythroblasts at different time points is quantified. Numbers represent at least 300 total cells per sample and error bars are SEM of three different independent experiments per stage. *p = 3.09e-6. (**d**) Images of primitive erythroblasts at the BasoE stage (E10.5) and the OrthoE stage (E12.5) in the microfluidic channel. Scale bar represents 20 µm. (**e**) Aspect ratio (D_*l*_/D_*s*_) changes of deforming primitive erythroid cells in the microfluidic channel. D_*l*_, cell length; D_*s*_, cell width. Time 0 indicates the time when cells enter the constriction channel. (**f**) Membrane mechanical stability of primitive erythroblasts was examined using fluorescence imaged microdeformation (FIMD). Brightfield (BF) and fluorescence images (Ter) of AlexaFluor 488-Ter119-labeled erythroid cell deformation. Left panel: In FIMD images of mechanically stable cells, the edges of cell boundary in brightfield (lipid bilayer) coincide with the distribution of Ter119 fluorescence (presence of the membrane cytoskeleton). Right Panel: Mechanical failure of the membrane cytoskeleton was indicated by the formation of skeletal-free regions that lack Ter119 fluorescence at the tip of the micropipette aspirated cell projection in FIMD images. (**g**) Frequency of maturing primitive erythroblasts and adult RBCs exhibiting membrane cytoskeletal failure in FIMD analysis. *p = 0.0017, **p = 9.53e-7. RBCs, adult red blood cells.

It was recognized more than a century ago that two overlapping populations of primitive and definitive erythroid cells circulate in the fetal bloodstream^5^. We have previously determined that primitive erythropoiesis in the mouse embryo first emerges as a transient wave of colony-forming progenitors in the E7.5-E9.0 yolk sac that generate a single cohort of circulating erythroblasts that progressively mature in the bloodstream^6, 7^. Morphologic examination of these cells indicate that they progress from immature proerythroblasts (ProE) at E9.5 to basophilic erythroblasts (BasoE) at E10.5 and ultimately to late-stage orthochromatic erythroblasts (OrthoE) by E12.5 (Fig. 1a). These late-stage primitive erythroblasts subsequently enucleate between E12.5-E16.5^7, 8^. The primitive erythroid lineage is superseded by definitive erythrocytes that emerge from the fetal liver beginning at E11.5-E12.5^9^, and rapidly become the predominant erythroid population in the fetal bloodstream^7^.

Maturing primitive and definitive erythroblasts share many features, including a progressive decrease in cell size, the accumulation of hemoglobin, nuclear condensation and, ultimately, enucleation to form mature erythrocytes. However, there are also significant differences between these lineages, including cell size, hemoglobin content, and the predominant expression of embryonic versus adult hemoglobins^10, 11^. Importantly, primitive erythroblasts mature intravascularly, unlike definitive erythroblasts that mature extravascularly before entering the bloodstream as reticulocytes. We have recently determined that late stage primitive erythroblasts in the E12.5 mouse embryo are highly deformable^12^, suggesting that they form a functional membrane skeleton prior to enucleating to cope with the vicissitudes of the fetal circulation. However, it is not known when during embryogenesis this functional network is established. In addition, little is known about the components that contribute to the membrane skeleton in primitive erythroblasts. Several genes known to be important constituents of the erythroid-specific membrane skeleton of definitive erythrocytes have been identified in late-stage primitive erythroblasts, specifically α-spectrin, β-spectrin, band 3 and ankyrinR^13, 14^. Actin and α-spectrin have also been localized to the cortex of E12.5 primitive erythroblasts^13^. To our knowledge, it is not known if primitive erythroid cells express other cytoskeleton-associated genes or if they express and specifically splice *Epb41* in an erythroid-specific manner.

Here, we report that comparably staged primitive and definitive erythroblasts express membrane skeleton-associated genes in a strikingly similar pattern as they mature. Given the necessity of circulating primitive erythroblasts for the survival of murine embryos beyond E10.5^15^, it was surprising to find that primitive erythroblasts at E10.5 are spherical and have minimal deformability. However, the transition of primitive erythroblasts over the subsequent 48 hours (i.e., between E10.5 and E12.5) to late-stage erythroblasts is characterized by changes in shape, the development of a highly deformable membrane, increased association of the membrane skeleton to the lipid bilayer, and the alternative splicing of *Epb41* exon 16. The importance of protein 4.1R in primitive erythropoiesis is evidenced by the spherocytic morphology and decreased membrane stability and deformability of late-stage *Epb41*-null primitive erythroblasts. Our findings indicate that immature primitive erythroblasts do not require a deformable membrane skeleton to successfully navigate the fetal bloodstream in the murine embryo before midgestation. In addition, protein 4.1R isoform switching and the development of a functional, protein 4.1R-dependent membrane skeleton are conserved in the primitive and definitive erythroid lineages of mammals.

## Results

### Primitive erythroblasts become deformable and develop a mechanically-stable cytoskeleton only at late stages of maturation

An erythroid-specific membrane skeleton determines the shape, mechanical stability, and membrane deformability of definitive red blood cells. Having previously determined that primitive erythroblasts at E12.5, which are at the OrthoE stage of maturation, are highly deformable^12^, we asked when during murine embryogenesis does this membrane skeleton become functional. Since the development of membrane skeleton function is associated with changes in the shape of adult red cells, we first examined the shape of primary primitive erythroblasts isolated at progressive days of embryogenesis. Virtually all primitive erythroblasts at E10.5 were spherical in shape (Fig. 1b,c). Strikingly, the population of the spherical primitive erythroblasts decreased sharply 24 hours later in development, so that almost all of the primary primitive erythroblasts at E11.5 and E12.5 display a partially concave shape (Fig. 1b,c). These data suggested that a deformable membrane is established as primitive erythroblasts undergo terminal maturation.

To directly assess the deformability of primitive erythroblasts, circulating cells from E10.5 and E12.5 mouse embryos were isolated and placed in dynamic flow channels. Primitive BasoE at E10.5 maintained their spherical shape and failed to deform in flow (Fig. 1d,e). In contrast, the partially concave primitive OrthoE at E12.5 elastically extended in flow, taking on a parachute-like shape reminiscent of human erythrocytes in capillaries due to tank treading (Fig. 1d,e). Primitive OrthoE gradually recovered their shape upon discharge from the channel. These findings indicate that a deformable membrane is specifically established only at late stages of primitive erythroblast maturation.

**Figure 2:**
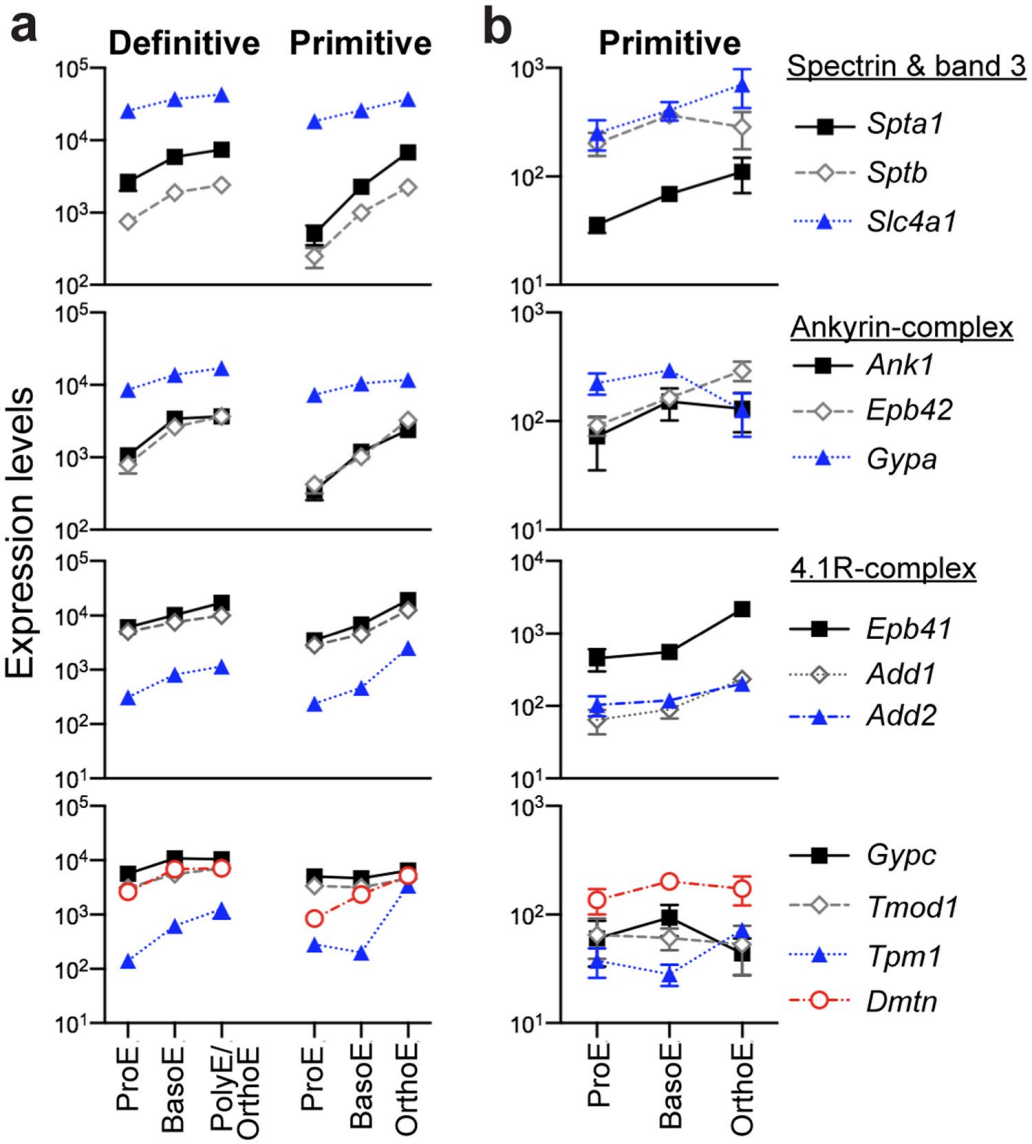
Major cytoskeletal genes are already expressed in ProE and transcript accumulation increases during maturation of primary murine primitive and definitive erythroid cells. (**a**) Relative transcript levels of cytoskeletal genes in primary definitive (left panel) and primitive (right panel) erythroblasts isolated from adult bone marrow and from staged embryos (ErythronDB: www.cbil.upenn.edu/ErythronDB)^16^. (**b**) Expression of cytoskeletal genes in purified maturing primitive erythroblasts was examined using quantitative real-time PCR. At least three independent experiments were performed at each gestational stage. Error bars represent SEM.

The erythroid membrane skeleton is anchored to the lipid bilayer to prevent cells from damage and rupture as they undergo shear stress and deformation. The mechanical stability of cells under extension was examined using fluorescence imaged microdeformation (FIMD), where TER119 labeling facilitates tracking the distribution of the membrane-associated skeleton in micropipette-aspirated cells. A proportion of erythroblasts exhibited skeletal-free regions at the tip of the projection within the pipette, representing separation of the skeletal network from the lipid bilayer (Fig. 1f, right panels). Essentially all primitive BasoE formed skeletal-free membrane regions, even at modest extensions (Fig. 2g, E10.5). The frequency of membrane separation significantly decreased in primitive PolyE at E11.5 and further declined in primitive OrthoE at E12.5, though they did not reach the frequency of fully mature adult red blood cells (Fig. 1g). These results indicate that there is a progressive strengthening of the association of the membrane skeleton with the lipid bilayer at late stages of primitive erythroid maturation.

### The kinetics of cytoskeletal gene expression are similar in primitive and definitive erythroblasts as they mature *in vivo*

While the composition of the membrane skeleton in definitive erythrocytes has been intensely investigated, surprisingly little is known about this network in primitive erythroid cells. We therefore asked if the genes representing the major components of the membrane skeleton in definitive erythroid cells are expressed in primary primitive erythroid cells. To establish a baseline for comparison, we first analyzed the kinetics of expression of 13 genes that constitute the spectrin-based backbone, as well as the major components of the ankyrinR and 4.1R complexes, in primary definitive erythroblasts isolated from the bone marrow of adult mice^16^ (ErythronDB). As shown in Fig. 2a (left panels), all of these genes encoding for proteins associated with the membrane skeleton are already significantly expressed in ProE and their expression uniformly increased as cells transition from ProE to BasoE in the bone marrow. As expected for the most abundant protein in the red cell membrane, band 3, encoded by *Slc4a1*, was the most abundant of the transcripts detected. Consistent with their importance in the function of mature red blood cells, the expression of all these genes either continued to increase or remained high in late-stage adult erythroblasts (PolyE/OrthoE; Fig. 2a, left panel). Similar results were evident from an independently established dataset of marrow-derived murine erythroblasts^17^ (Supplementary Fig. S1).

We next analyzed the expression of the major membrane skeleton genes in purified populations of primitive erythroblasts at sequential days of embryogenesis. All 13 genes expressed by definitive erythroblasts, including components of the spectrin backbone (α1- and β1-spectrin), the ankyrinR complex (ankyrinR, protein 4.2, and glycophorin A), and the protein 4.1 R complex (protein 4.1R, α-adducin, β-adducin, glycophorin C, tropomodulin 1, tropomyosin 1, and dematin) are also expressed in primary primitive erythroblasts (Fig. 2a, right panels). All of these genes were already expressed at significant levels in E9.5 primitive ProE, with band 3 (*Slc4a1*) being the most abundant. These genes were subsequently all upregulated as primitive erythroblasts mature *in vivo*. Comparison with definitive erythroblasts reveals a strikingly similar pattern and levels of gene expression during maturation (Fig. 2a).

To further examine and validate the expression of major cytoskeletal genes in primitive erythroblasts, we employed qPCR with samples of primitive erythroblasts independently isolated by fluorescence activated cell sorting (FACS) from E9.5, E10.5, and E12.5 mouse embryos. Transcripts of all 13 cytoskeletal genes were detected in primitive ProE (E9.5), increase in primitive BasoE (E10.5), and continue to be expressed in primitive OrthoE (E12.5) (Fig. 2b). Overall, these qPCR data confirm the expression data derived from the microarray analysis (Fig. 2a). Taken together, our data indicate that maturing primitive erythroblasts express the major genes found in the erythroid-specific membrane skeleton of maturing definitive erythroblasts. Their strikingly similar kinetics of expression suggest that the development of an erythroid-specific membrane skeleton might be conserved in primitive and definitive erythropoiesis.

### Cytoskeletal proteins associate with the plasma membrane only at late stages of primitive erythroblast maturation

**Figure 3:**
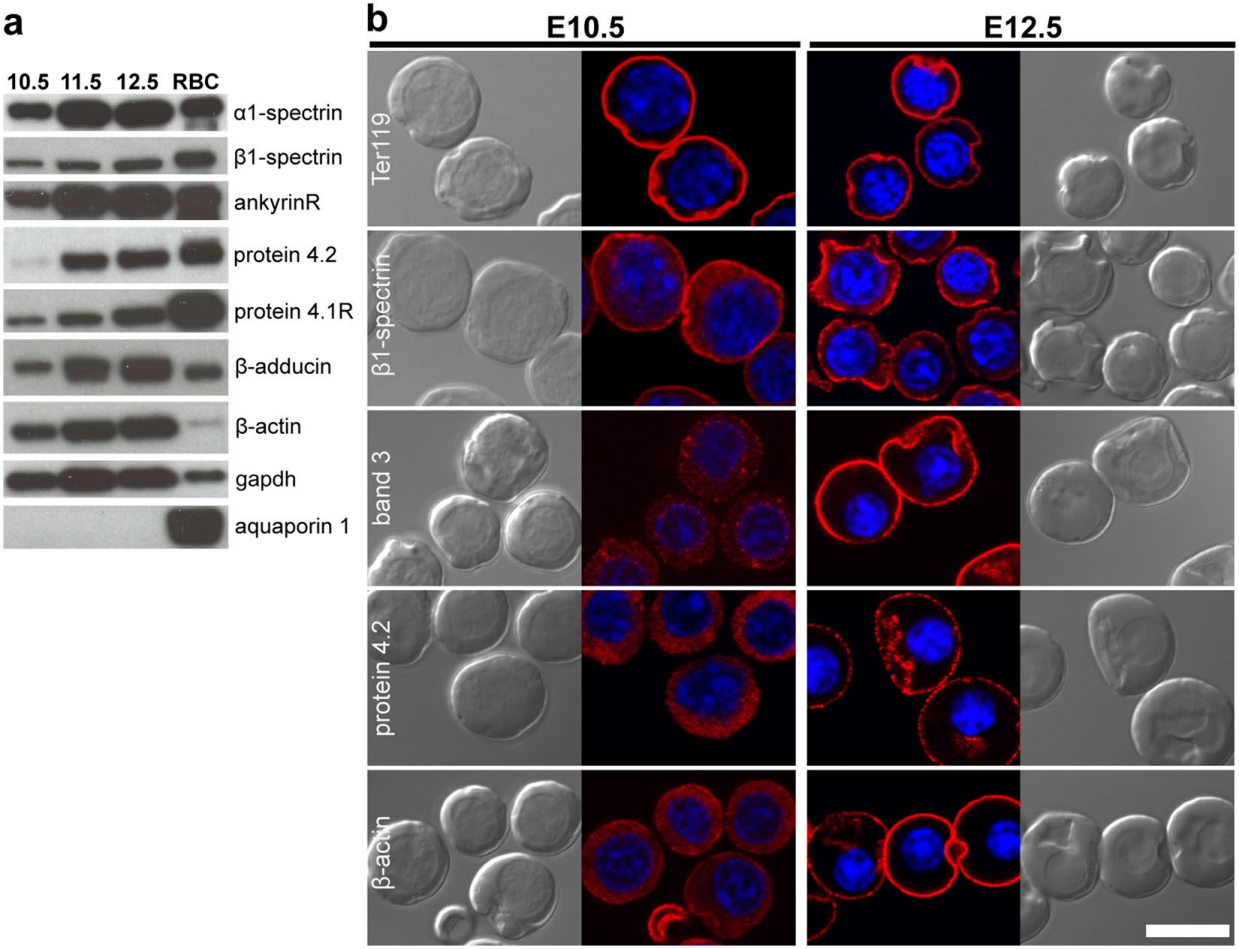
Expression of membrane skeleton proteins in primary primitive erythroblasts. (**a**) Immunoblots of whole cell lysates prepared from 5 × 10^5^ cells loaded per lane. RBC, adult RBCs. Representative data from one of two or three independent experiments are shown. (**b**) Immunofluorescence of cytoskeletal proteins (red) in primitive erythroblasts from E10.5 (left panels) and E12.5 (right panels) mouse embryos. Parallel brightfield and fluorescence images are shown. Nuclei were stained with Hoechst (blue). Band 3, protein 4.2, and β-actin become localized to the plasma membrane only at late stages of maturation. Representative data from one of three independent experiments are shown. Scale bar represents 10 µm.

The acquisition of a functional membrane skeleton in primitive erythroblasts between E11.5 and E12.5 suggests that membrane skeleton proteins become associated with the membrane only at late stages of maturation. We therefore examined the accumulation of cytoskeletal proteins and their cellular distribution during progressive stages of primitive erythroblast maturation. As shown in Fig. 3a, several cytoskeletal proteins, including α1- and β1-spectrin, ankyrinR, protein 4.1R, protein 4.2, and β-adducin, were detected already in whole cell lysates of primitive BasoE (E10.5), and increased in amount as cells transition to PolyE/OrthoE (E12.5). Notably, protein 4.1R in primitive erythroblasts co-migrated with the major ~80 kDa isoform detected in adult red cells, while no expression of larger 135 kDa isoforms, normally found in many non-erythroid tissues, was observed. Aquaporin 1, which is expressed specifically in definitive, but not primitive, erythroid cells^16^, indicated that the embryonic blood samples were not significantly contaminated with maternal red blood cells.

We utilized immunofluorescence to investigate the subcellular localization of membrane skeleton proteins in maturing primitive erythroblasts. Unfortunately, only a small number of available antibodies were determined to be useful in immunohistochemical studies. As shown in Fig. 3b, glycophorin A (Ter119) and β1-spectrin were already localized to the plasma membrane of primitive BasoE at E10.5, while band 3, protein 4.2, and actin were localized predominantly in the cytoplasm (Fig. 3b, left panels). However, these latter cytoskeletal proteins subsequently become localized to the plasma membrane as the cells transition to OrthoE at E12.5 (Fig. 3b, right panels). These data suggest that the initial membrane contains β1-spectrin and glycophorin A and that an erythroid-specific cytoskeleton containing band 3 subsequently forms at late stages of maturation.

### Primitive erythroblasts undergo *Epb41* alternative splicing in a narrow maturational window

**Figure 4:**
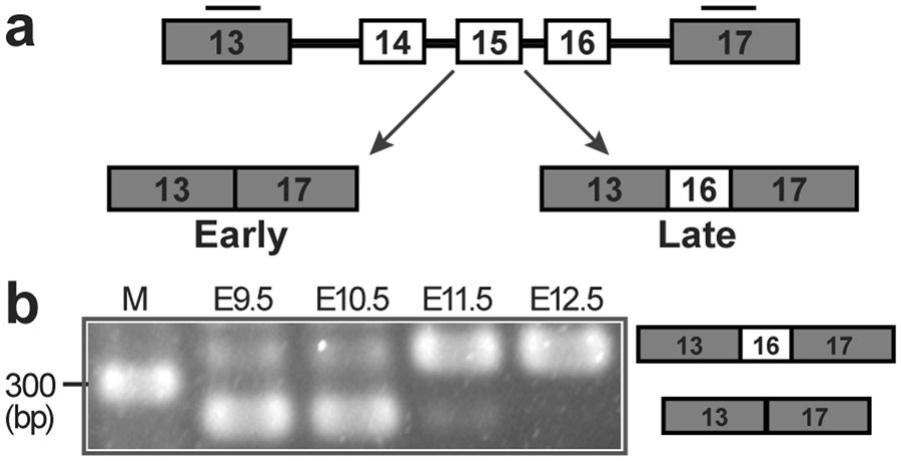
Primitive erythroblasts undergo developmental-specific alternate splicing of *Epb41* as they transition from BasoE at E10.5 to PolyE at E11.5. (A) *Epb41*, encoding protein 4.1 R, undergoes maturation-specific alternative splicing in definitive erythropoiesis. *Epb41* exon 16 encodes a high spectrin-affinity domain and is exclusively incorporated into *Epb41* transcripts in definitive erythroblasts at late maturational stages. Bars represent the location of the primer pairs used to detect *Epb41* isoforms by RT-PCR. Constitutive exons are gray and alternatively spliced exons are white. (**b**) Splice variants of *Epb41* in primitive erythroblasts during maturation were detected by RT-PCR. A representative analysis of 3 independent experiments is shown. Exons included in the bands are indicated in the right panel. M, DNA ladder.

Protein 4.1R, encoded by *Epb41*, undergoes alternative splicing in maturing definitive erythroid cells (Fig. 4a). The erythroid-specific protein 4.1R isoform, protein 4.1R^80^, contains exon 16, which encodes for a spectrin/actin-binding domain^3, 18^. We asked whether the erythroid-specific *Epb41* alternative splicing also occurs in primitive erythropoiesis. Exon 16 was essentially excluded from *Epb41* transcripts in primitive erythroblasts at E9.5 and E10.5, but was highly expressed in circulating primitive erythroblasts isolated from E11.5 and E12.5 mouse embryos (Fig. 4b). Since protein 4.1R undergoes isoform switching in definitive erythroid cells, we examined maturing primitive erythroblasts from E9.5, E10.5 and E12.5 mouse embryos for differential exon usage by RT-PCR. No significant evidence of alternative splicing outside of the spectrin/actin-binding domain was found, suggesting no heterogeneity in the N-terminal FERM domain or C-terminal domain that interact with other red cell proteins. Consistent with immunoblots showing expression of 80 kDa protein 4.1R (Fig. 3a), this transcript analysis detected mainly isoforms initiated at exon 1A, previously shown to splice so as to encode only 80 kDa protein^19^ (Supplementary Fig. S2). Taken together, these data indicate that *Epb41* isoform switching of exon 16 is a feature of primitive erythropoiesis and occurs in a narrow developmental window as primitive BasoE transition to PolyE.

**Figure 5:**
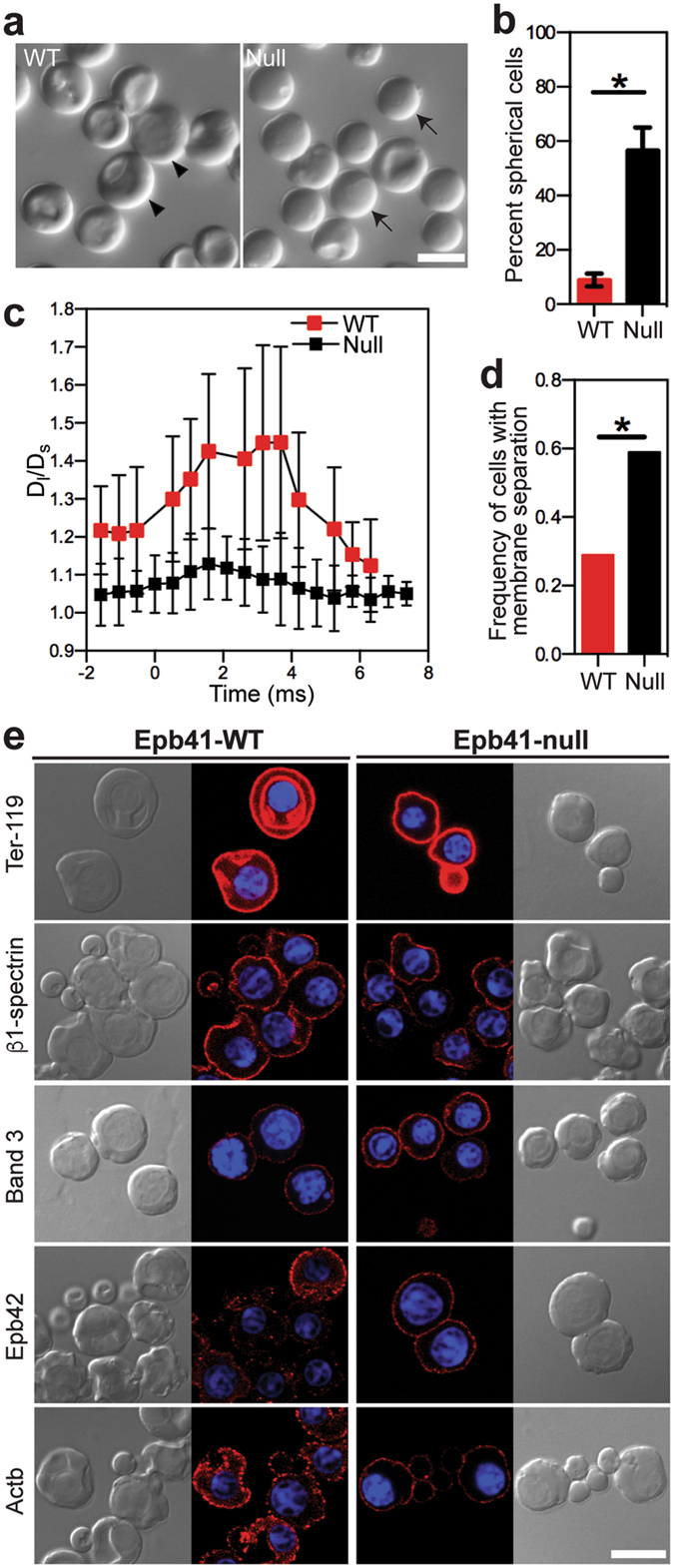
*Epb41*-null primitive erythroblasts at the OrthoE stage (E12.5) have abnormal cell shape and defects in physical membrane features. (**a**) Morphology of freshly isolated *Epb41*-wild-type (WT) and *Epb41*-null primitive erythroblasts at E12.5. Spherical cells, arrows; partially concave cells, arrow heads. (**b**) The percentage of spherical cells at E12.5. Numbers represent at least 300 total cells per sample and error bars are SEM of three independent samples per stage. *p = 0.0028. (**c**) Aspect ratio (D_*l*_/D_s_) changes of deforming E12.5 *Epb41*-WT and *Epb41*-null primitive erythroid cells in the microfluidic channel. D_*l*_, cell length; D_s_, cell width. Time 0 indicates the time when cells enter the constriction channel. (**d**) AlexaFluor 488-Ter119 labeled E12.5 *Epb41*-WT and *Epb41*-null primitive erythroblasts were analyzed using fluorescence imaged deformation (FIMD). A significantly higher frequency of *Epb41*-null primitive OrthoE display evidence of membrane separation. *p = 0.0212. (**e**) Immunofluorescence of cytoskeletal proteins (red) in *Epb41*-WT and *Epb41*-null E12.5 primitive erythroblasts. Nuclei were stained with Hoechst (HO). Scale bar represents 10 µm.

The *Epb41* isoform switch in primitive erythroblasts correlates temporally with changes in cell shape, cellular deformability, mechanical stability, and localization of proteins to the plasma membrane, suggesting that protein 4.1R plays a functional role in the cytoskeletal network. While we could not test the specific role of exon 16, we did examine the structure and function of circulating primitive erythroblasts from *Epb41*-null mice. Unlike their wild-type counterparts, a large proportion of *Epb41*-null primitive OrthoE at E12.5 failed to transition from spherical to partially concave shape (Fig. 5a,b). Importantly, E12.5 *Epb41*-null OrthoE were not deformable in dynamic flow channels (Fig. 5c), reflecting a reduced area to volume ratio. Additionally, E12.5 *Epb41*-null primitive erythroblasts underwent an increased frequency of lipid bilayer separation in FIMD studies, consistent with a failure to form a mechanically stable membrane (Fig. 5d). We then examined if the lack of protein 4.1R affects the localization of other membrane skeleton proteins at the plasma membrane. Consistent with their adult counterparts^20, 21^, E12.5 *Epb41*-null primitive erythroblasts still localize β1-spectrin, glycophorin A, and band 3 to the plasma membrane (Fig. 5e). Taken together, these data indicate that *Epb41*-null primitive erythroid cells fail to form a functional membrane skeleton network prior to their enucleation despite the localization of several proteins to the plasma membrane.

## Discussion

**Figure 6:**
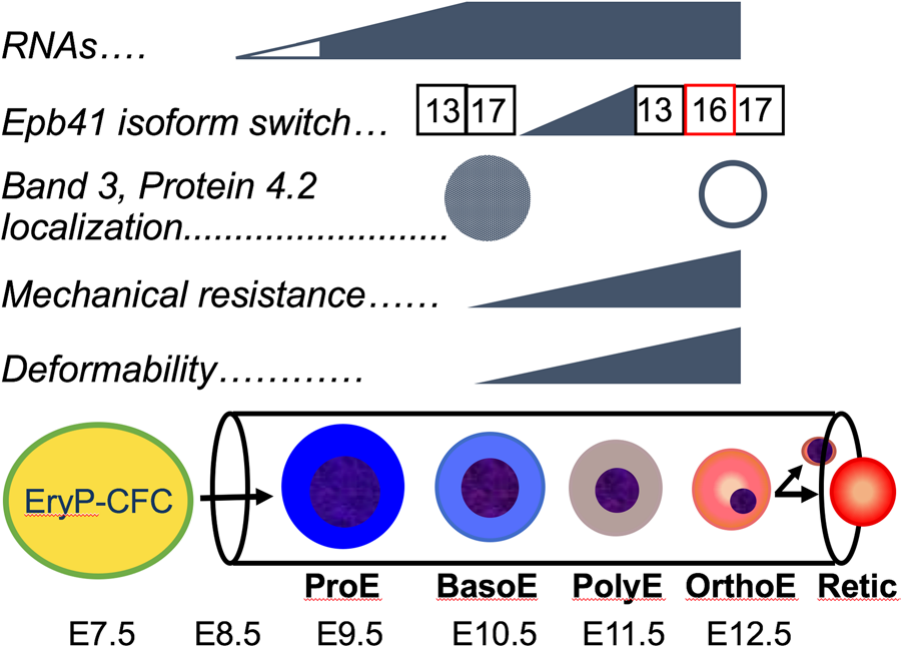
Model of kinetics of membrane skeleton network formation in primary primitive erythroblasts. Primary primitive erythroblasts progressively and synchronously mature between E9.5 and E12.5 in the bloodstream of the mouse embryo prior to enucleating. The expression of the major cytoskeletal genes found in definitive red blood cells has already initiated in primary primitive proerythroblasts (ProE) at E9.5. The cytoskeletal proteins β1-spectrin, band 3, and protein 4.2 transition to the plasma membrane at late stages of erythroblast maturation concomitant with the alternative splicing of *Epb41* exon 16. This transition is associated with significant increases both in the association of the cytoskeleton to the lipid bilayer and in cell deformability. BasoE- basophilic erythroblast, PolyE- polychromatophilic erythroblast, OrthoE- orthochromatic erythroblast, Retic- reticulocyte.

The cardiovascular system, including primitive erythroid cells, blood vessels, and a beating heart, is the first functional organ system in the mammalian embryo^22^. The primitive erythroid lineage emerges as a transient wave of progenitors in the yolk sac beginning at E7.25^6^ (Fig. 6). With the onset of cardiac contractions at E8.25, primitive erythroblasts begin to leave yolk sac blood islands and circulate in the newly formed vasculature of the yolk sac and the embryo proper^23^. Their numbers rapidly expand so that primitive erythroid cells constitute approximately 45–50% of the cellular mass of the E10.5 mouse embryo, the time when a robust circulation is established^23, 24^. Importantly, primitive erythroblasts contribute to the hemodynamic forces necessary for remodeling of the yolk sac vascular plexus into an arborized network between E9.5-10.5, which coincides with an increase in shear stress in the vasculature^25, 26^. The specific absence of maturing primitive erythroblasts, as occurs following targeted disruption of Gata1, leads to failure of vascular remodeling and to fetal demise between E10.5-E11.5^15^. These findings highlight the central importance of maturing primitive erythroblasts both for normal cardiovascular development and survival of the murine embryo prior to the emergence of definitive red blood cells.

Given the necessity of circulating primitive erythroblasts at E9.5–10.5, we asked whether these immature cells require a deformable cytoskeleton to navigate the early embryonic vasculature. Surprisingly, we determined that primary primitive erythroblasts at E10.5 are spherical in shape, lack a deformable membrane when placed in flow conditions, and have poor membrane skeleton-lipid bilayer association. These data indicate that immature primitive erythroblasts are able to successfully navigate the fetal bloodstream and contribute to the shear stress necessary for vascular remodeling and embryonic survival despite lacking a deformable cytoskeleton.

Using functional studies we determined that primitive erythroblasts establish a deformable membrane skeleton associated with the lipid bilayer only at late stages of maturation. To shed light on the potential components of this membrane skeleton in primitive erythroblasts, we examined the expression of 13 genes comprizing essential components of the erythroid-specific membrane network of definitive red blood cells. All of these genes, including components of the spectrin backbone, the ankyrinR complex, and the protein 4.1R junctional complex are also expressed in primary primitive erythroblasts. The kinetics of transcript accumulation of these membrane skeletal genes was surprisingly similar in morphologically comparable stages of primitive and definitive erythroblasts, suggesting that the programs responsible for formation of the erythroid-specific membrane skeleton are conserved in primitive and definitive erythropoiesis. It would be of interest to determine if a similar program exists in the primitive and definitive erythroid lineages of non-mammalian species that do not enucleate^27, 28^.

The formation of the membrane skeletal network has primarily been studied using *in vitro* model systems as surrogates for *in vivo* events^29–33^. The synchronous maturation of primitive erythroblasts provides a unique opportunity to investigate the *in vivo* establishment and function of the cytoskeletal network in primary erythroblasts at progressive stages of maturation. Our studies reveal the upregulation of several cytoskeletal genes in ProE, followed by the accumulation of band 3 and protein 4.2 in the cytoplasm of BasoE and the transition of these proteins to the cell membrane in late stage erythroblasts (Fig. 6). Studies of human erythroblasts differentiated *in vitro* reveal a similar cascade of events^32, 34^. We find that these events lead to a deformable membrane skeleton in primary primitive erythroblasts. However, to our knowledge, functional studies of deformability or membrane skeleton-lipid bilayer association have not been performed with definitive erythroblasts at progressive stages of maturation.

A functional cytoskeletal network in definitive red blood cells requires protein 4.1R, which undergoes an isoform switch incorporating exon 16, which encodes an evolutionarily conserved domain that mediates the high affinity binding of protein 4.1R to β-spectrin^35, 36^. The establishment of a functional membrane skeletal network in maturing primitive erythroblasts coincides with the erythroid-specific splicing of *Epb41* (Fig. 6). While the timing of the alternative splicing of *Epb41* exon 16 in definitive erythroblasts has not been established^3, 37^, this splicing event occurs in a narrow BasoE to PolyE maturational window in primitive erythropoiesis.

Targeted disruption of the junctional protein, dematin, leads to anemia at birth and severe hemolytic anemia in adult mice^38^, providing evidence for the functional importance of the junctional complex in definitive erythropoiesis. In contrast, the normal morphology of E13.5 dematin-null primitive erythroblasts led to the speculation that dematin lacks a role in primitive erythropoiesis^38^. However, functional studies of primitive erythroblasts were not performed. Using both functional FIMD and flow-based studies, we found that E12.5 *Epb41*-null primitive erythroblasts lack mechanical strength and have markedly reduced cellular deformability, despite the localization of several cytoskeletal proteins at the plasma membrane. Our findings indicate that protein 4.1R plays an important functional role not only in definitive, but also in primitive, erythropoiesis and highlights the conservation of an erythroid-specific program during mammalian ontogeny.

## Methods

### Mice and collection of embryonic blood

All animal experiments were approved by the University of Rochester Committee on Animal Resources and were performed in accordance with the relevant institutional guidelines and regulations. ICR (Taconic Biosciences), *Epb41*-heterozygous and *Epb41*-null mice^20^ were mated overnight and vaginal plugs checked the following morning (E0.3). Time-pregnant mice were sacrificed by CO_2_ narcosis and embryos dissected in PB2^16^, containing 12.5 μg/mL heparin. *Epb41* embryos were genotyped using the AccuStart II Mouse Genotyping Kit (Quanta Biosciences) using primer pairs *for Neo* and *Epb41* exon 4^20^. E9.5 yolk sacs were dissociated with 0.5 mg/mL collagenase Type IV (Stem Cell Technologies) in PB2 at 37 °C. Peripheral blood was collected from E9.5-E12.5 embryos as described^7^.

### Gene expression analysis

Expression of cytoskeletal genes in primary primitive erythroblasts was determined by quantitative RT- PCR (qPCR), as previously described^16^, using primer pairs listed in Supplementary Table S1 and normalized to 18S. The following primer pairs and GoTaq Hot Start Polymerase (Promega) were used to distinguish *Epb41* alternative splicing: Epb41E13-f, 5′-CTGAGTCCACAGACCGAAGTC-3′; Epb41E17-r: 5′-CGGGTACCGATTCCATAAAGT-3′^39^. The identity of *Epb41* exon 16-related PCR products, shown in Fig. 4, was confirmed by sequence analysis. Qualitative RT-PCR analysis of protein 4.1R exon usage was performed with primer pairs listed in Supplementary Table S2, as described in Supplementary Methods.

### Preparation of primitive erythroid cell lysates

Primitive erythroblasts were washed 3 times with PBS and whole-cell lysates prepared in RIPA buffer in the presence of complete protease inhibitor cocktail (Roche), 25 μg/mL PMSF (Thermo Fisher Scientific), and 1 mM Na_3_VO_4_. Nuclei were separated by centrifugation at 500 *g* for 10 minutes at 4 °C. One-third volume 3X sample storage buffer (3X SSB, 187.5 mM Tris, 6% SDS, 30% glycerol, 3 mM DTT) was added and samples stored at −80 °C until analysis.

### Immunoblotting and Immunofluorescence analyses

Antibodies included monoclonal anti-β-actin (Sigma-Aldrich, A1978), anti-Ter119 (BD Pharmingen, 553671), anti-β1-spectrin (Santa Cruz Biotechnology, SC-7466), and anti-AQP1 (Santa Cruz Biotechnology, SC-20810). All remaining antibodies were in our laboratories^21, 40^. Secondary antibodies were HRP-conjugated (BioRad) for immunoblotting, and AlexaFluor647-conjugated (Thermo Fisher Scientific) for immunofluorescence.

For immunoblotting, cell lysates prepared from 5 × 10^5^ primitive erythroblasts or adult red blood cells were separated by 4–20% SDS-PAGE (Bio-Rad) and transferred to PVDF membranes (EMD Millipore). Membranes were blotted with antibody in 5% (w/v) nonfat dry milk (Bio-Rad) in TBST (50 mM Tris, 150 mM NaCl; 0.1% Tween-20), and washed in TBST. Signal was detected using the Pierce ECL Plus Western Blotting Substrate (Thermo Scientific) and developed on BioMaxXAR film (Carestream/Kodak).

For immunofluorescence, primitive erythroblasts were pelleted at 1,100 *g* for 4 minutes and fixed by sequentially adding 50–100 μl of 0.5% acrolein (Sigma-Aldrich or Alfa Aesar) in PBS for 5 minutes, equal volume of 2X formaldehyde buffer (2X PBS containing 4% formaldehyde, [Polyscience Inc], and 200 mM glycine) for 5 minutes, and SLAT-H buffer^7^ for 5 minutes. Cells were cytospun onto Bond-Rite slides (Thermo Fisher Scientific), washed three times in rinsing buffer (100 mM glycine in PBS), permeabilized with 0.05% Triton X-100 for 10 seconds, washed three times in rinsing buffer, and incubated in rinsing buffer for 30 minutes. Cells were incubated in blocking buffer (PBS containing 0.2% fish skin gelatin, [Sigma-Aldrich], and 50 mM glycine) for 1 hour, stained with antibodies diluted in blocking buffer, and washed in rising buffer three times after each staining. Cells were stained in 1 μm/ml of Hoechst 33342 (HO, Sigma-Aldrich) for 5 minutes and mounted in ProLong Gold antifade reagent (Thermo Fisher Scientific). Images were acquired with TCS SP5 confocal microscope system (Leica) with 100X/1.44 oil immersion objective (Leica). At least three independent samples were analyzed at each gestational age.

### Morphologic analyses

Cells were cytospun (Shandon II, Thermo Fisher Scientific), Wright-Giemsa stained, permounted and coverslipped. Images were acquired on a DS-Fi1 camera (Nikon) attached to an Eclipse 80i microscope (Nikon) with a using 40X/0.75 objective.

To examine the morphology of living cells, 10 μl of embryonic blood was loaded between two cover slips mounted onto a glass slide and covered with a third cover slip. Images were acquired with an ORCA-R2 digital camera (Hamamatsu) and NIS-Elements software on an Eclipse 80i microscope (Nikon) with a 40X/0.75 objective. Photoshop CS5 software (Adobe) was used for image processing.

### Microfluidic evaluation of cell deformability

Microchannels were fabricated using standard techniques of soft photolithography in polydimethylsiloxane (PDMS). All channels were 100 μm(w) × 22 µm(h) containing a single 100(lc) × 20(wc) µm constriction. Cells were centrifuged at 300 *g* for 1 minute at room temperature, resuspended in PB2, and diluted in physiological salt solution (PSS, 4.7 mM KCl, 2.0 mMCaCl_2_, 1.2 mM MgSO_4_, 140.5 mM NaCl, 21 mM Tris, 11.1 mM dextrose, pH7.4) containing 0.1% BSA. High-speed videos were acquired at ~1900 frames/sec with a 30μs exposure with a Phantom Miro M120 camera (Vision Research) on a DMIRB microscope system (Leica) with a 63 × / oil immersion objective. Movies were analyzed using ImageJ^41^. At least three independent experiments were performed and more than 30 cells were analyzed at each gestational age.

### Fluorescence imaged microdeformation (FIMD)

FIMD was used to determine the frequency of lipid bilayer separation from the cytoskeletal network^42^. Briefly, collected erythroid cells were suspended in blocking buffer (4% plasma-derived serum [PDS; Animal Technologies] prepared in PBS [300 mOsm]) at 37 °C for 15 minutes, stained with 1:100 AlexaFluor 488-Ter119 (BioLegend) at 37 °C for 15 minutes, and washed once with the blocking buffer. Cells were aspirated into a glass micropipette with 2–4 μm diameter at approximately 1,500 Pa (15 cm H_2_O) pressure. Care was taken to ensure the absence of membrane folds. Images were acquired with QuantEM:512SC EMCCD camera (Roper Scientific) with NIS-Elements software on an Eclipse TE2000 microscope (Nikon) with a 40 × /0.75 objective and analyzed using ImageJ. The membrane projection lengths in the pipette, *L*_*p*_, and the pipette diameter, *R*_*p*_, were measured in brightfield images; cells with extension ratios (*L*_*p*_ */R*_*p*_) between 5-12.5 were analyzed. Separation of the cytoskeletal network from the lipid bilayer was observed as differences in the extension lengths of fluorescently-labeled cytoskeleton and membrane projections in brightfield. At least three independent experiments were performed and more than 25 cells were analyzed at each gestational stage.

### Statistics

Statistical analysis was performed by 2-tailed Student’s *t*-test, except for the FIMD analysis where the z-test was applied.

## Electronic supplementary material

Supplementary Information
