# Supplementary material for: Circulating primitive erythroblasts establish a functional, protein 4.1R-dependent cytoskeletal network prior to enucleating
Source: Sci Rep. 2017 Jul 12;7:5164. doi: 10.1038/s41598-017-05498-4 (PMC5507979; doi:10.1038/s41598-017-05498-4)
Supplement: Supplementary file 1 — Supplementary Information [file 41598_2017_5498_MOESM1_ESM.pdf]

Supplementary Information

Circulating primitive erythroblasts establish a functional, protein 4.1R-dependent cytoskeletal network prior to enucleating

Authors

Yu-Shan Huang[1], Luis F. Delgadillo[2], Kathryn H. Cyr[3], Paul D. Kingsley[4], Xiuli An[5], Kathleen E. McGrath[4], Narla Mohandas[5], John G. Conboy[6], Richard E. Waugh[2], Jiandi Wan[3,7], and James Palis[1,4,*]

[1]Department of Biomedical Genetics, [2]Department of Biomedical Engineering, [4]Department of Pediatrics and Center for Pediatric Biomedical Research, University of Rochester, Rochester, NY 14642; [3]Department of Biomedical Engineering, [7]Microsystems Engineering, Rochester Institute of Technology, Rochester, NY 14623; [5]Red Cell Physiology Laboratory, New York Blood Center, New York, NY 10065; and [6]Life Sciences Division, Lawrence Berkeley National Laboratory, Berkeley, CA 94720

Supplementary Table 1. Primer pairs for qPCR analysis.

Genes	Forward	Reverse	Reference
Spta1	5'-CCA AGC TCT GGC AGA AGG CAA GGC-3'	5'-CCC ATT ACT TGC TGC CAC ACT ATG-3'	Modified from Nilson et al., 2006[1]
Sptb	5'-GCT TAA GGA ACG CCA GAC TCC AG-3'	5'-CAC ACC CTG TCC TCG TCC TGG CC-3'	Modified from Nilson et al., 2006[1]
Slc4a1	5'-CAC AGT GCC TCT CCG TCG TCT CAT C-3'	5'-CCT TCC CCA CCC ACA GCC ATA ACA C-3'	[1]
Ank1	5'-CCG TTG TGA TCC GAT CTG AAG-3'	5'-CAC AGG GCT AAT GTT GTC TGA G-3'	[2]
Epb42	5'-TTC CAC GCA GCA GAA AAC AAC-3'	5'- GCA CGG AAG TTC AGG GTG ATA G-3'	PrimerBank[3] ID: 7305035a1
Gypa	5'-GCC GAA TGA CAA AGA AAA GTT CA-3'	5'-TCA ATA GAA CTC AAA GGC ACA CTG T-3'	[4]
Epb41	5'-GCT CAG GAA GAA CAC AGA GAG G-3'	5'-CAT TCG TAG ACC GTG TCA TCC-3'	[5]
Add1	5‘-TGA AGA AGA CCT TCC CCA GGA GCC-3’	5'-GGG CGT CGG CTT GAA GGT GG-3'	Designed using Primer-BLAST[6]
Add2	5'-TGA CAC CCA TCA ACG ACC TC-3'	5'-CTG ATC TTG CAC CGC ATA AGC-3'	PrimerBank ID: 146134376c1
Gypc	5'-TGT GCC ACC ATT ATC AGC ACC-3'	5'-GAC TCC TCC CAT CTG TAT CGG-3'	PrimerBank[3] ID: 21313681a1
Tmod1	5'-CGA AGG AAT TTA AGG ACC GAG AA-3'	5'-CCC AGG ATA GCT GCG ATG TC-3'	PrimerBank[3] ID: 144922650c2
Tpm1	5'-TTG AAA GCC GAG CCC AAA AAG-3'	5'-TCAT ACT TCC GGT CAG CAT CTT-3'	PrimerBank[3] ID: 256000787c1
Dmtn	5'-GCA GAA GCA ACC TCT TAC CTC-3'	5'-AGA TCC TTG TAG CCC AAC ACC-3'	PrimerBank[3] ID: 7305037a1
Rn18s	5'-TTG ACG GAA GGG CAC CAC CAG-3'	5'-GCA CCA CCA CCC ACG GAA TCG-3'	RTPrimerDB[7] ID: 1014
Hbb-y	5'-CTC TAG CTG TCC AGC AAT CCT G-3'	5'-GCT TTC AAG GAA CAG TCC AGT ATT C-3'	[8]
Hbb-bh1	5'-AGT TTG GAA ACC TCT CTT CTG CCC TG-3'	5'-TGT TCT TAA CCC CCA AGC CCA AG-3'	[8]
Hbb-b1	5'-GCT CTT GCC TGT GAA CAA TG -3'	5'-GTC AGA AGA CAG ATT TTC AAA TG-3'	[8]

Supplementary Table 2. Primer pairs for PCR analysis of Epb41 exon usage.

Exon	Forward	Reverse	Product Size
1a	5'-CAG GTC CCT GTC CTG TGC-3'		363
1c	5'-ATT GTT CGT CGG GCT GAA GC-3'		411
2		5'-CCC CCT TCA CCT TTC TCT TT-3'	
2	5'-AAG GTG AAG GGG GTC AGA AG-3'		655
8		5'-AGG GCT AAA GTT GCA AAG GA-3'	
7	5'-CCG ACC CAG CAC AAT TAA CA-3'		903
13		5'-CCC GCT TCT CTT CAA CCT TC-3'	
17	5'-GAA TCG GTA CCC GAA CCA C-3'		409
20		5'-GGT CGA TAT CGG CAT CTC CT-3'	
19	5'-AGA CCC TGG AGT CTT GCT GA-3'		385
22		5'-TTG GTT TTC TGT GGT GTG GA-3'	

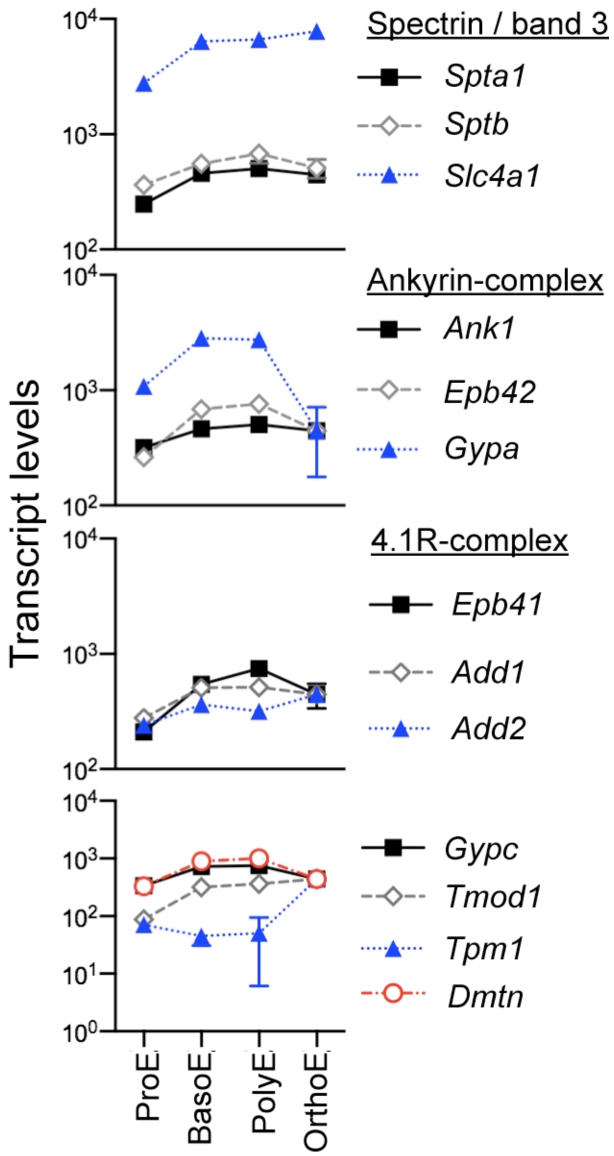

Supplementary Figure S1. Expression of genes associated with the cytoskeleton in bone marrow-derived erythroblasts at progressive stages of maturation. Comparison of transcript levels of cytoskeletal genes in definitive erythroblasts isolated from adult mouse bone marrow. Data derived from An, et al. 2014[10]. ProE, proerythroblasts; BasoE, basophilic erythroblasts; PolyE, polychromatophilic erythroblasts; and OrthoE, orthochromatic erythroblasts.

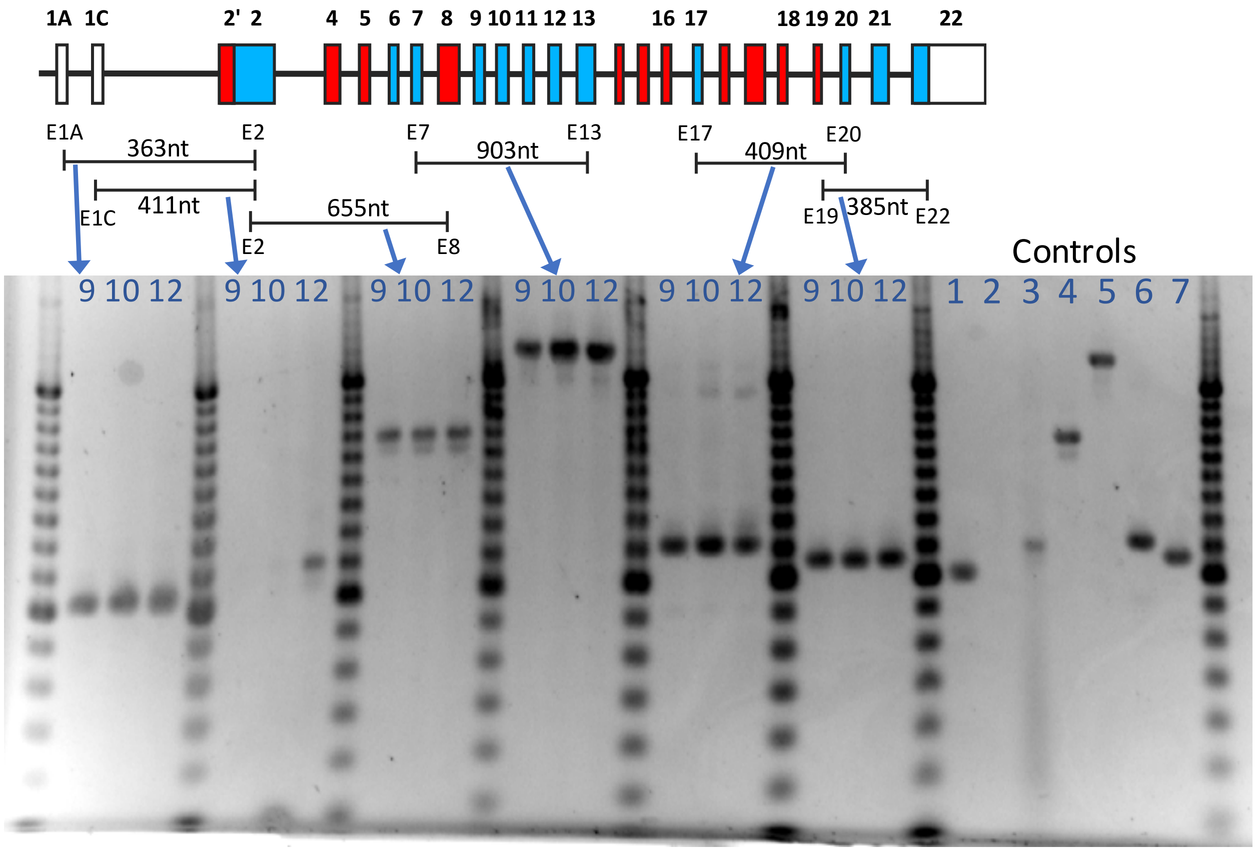

Supplementary Figure S2. RT-PCR analysis of *Epb41* exon usage during primitive erythroid maturation from E9.5 – E12.5. Transcript accumulation including exons 1c and 2' is observed at E12.5, but not E9.5 and E10.5. Other exon usage spanning the *Epb41* gene (with the exception of exon 16, Fig. 4) is unchanged. Controls: lane 1: E1A-E2, bone marrow (BM), lane 2: E1C-E2 BM, Lane 3: E1C-E2 brain, Lane 4: E2-E8 BM, Lane 5: E7-E13 BM, Lane 6: E17-E20 BM, Lane 7: E19-E22 BM. 50 bp ladder with bands from 50 – 800 bp. Red exons in the 4.1 gene diagram represent exons for which there is evidence of alternative splicing in human tissues. Blue exons are considered constitutive, while white exons represent noncoding regions.

Supplementary Methods:

RT-PCR analysis of *Epb41* exon usage.

Primitive erythroid cells were sorted from E9.5 (yolk sac and blood), E10.5 (blood) and E12.5 (blood) as previously described[9]. RNA was isolated using Qiagen RNeasy plus mini kit with gDNA eliminator. RNA was reverse transcribed with High Capacity Reverse Transcription kit with RNase Inhibitor (Applied Biosystems/ThermoFisher). PCR was performed on a BioRad C1000 thermocycler using Quanta Bio Accustart II PCR Supermix. All annealing was performed at 58°C, with the exception of Exon 1C-2 was performed at 64°C. Extensions were at 70°C for 30”, denaturing at 93°C for 20” (after an initial activation at 95°C for 2 min).

Supplementary References

1. Nilson, D.G., Sabatino, D.E., Bodine, D.M., Gallagher, P.G. Major erythrocyte membrane protein genes in EKLF-deficient mice. *Exp. Hematol.* **34,** 705–712 (2006).
2. Huang, H., et al. A deep intronic mutation in the ankyrin-1 gene causes diminished protein expression resulting in hemolytic anemia in mice. *G3 Bethesda MD*. **3,** 1687–1695 (2013).
3. Wang, X. & Seed, B. A PCR primer bank for quantitative gene expression analysis. *Nucleic Acids Res.* **31,** e154 (2003).
4. Basu, P., et al. KLF2 is essential for primitive erythropoiesis and regulates the human and murine embryonic beta-like globin genes in vivo. *Blood* **106,** 2566–2571 (2005).
5. Shi, Z.T., et al. Protein 4.1R-deficient mice are viable but have erythroid membrane skeleton abnormalities. *J. Clin. Invest.* **103,** 331–340 (1999).
6. Ye, J., et al. Primer-BLAST: a tool to design target-specific primers for polymerase chain reaction. *BMC Bioinformatics* **13,** 134 (2012).
7. Pattyn, F., Speleman, F., De Paepe, A. & Vandesompele, J. RTPrimerDB: the Real-Time PCR primer and probe database. *Nucleic Acids Res.* **31,** 122–123 (2003).
8. Kingsley, P.D., et al. “Maturational” globin switching in primary primitive erythroid cells. *Blood* **107,** 1665–1672 (2006).
9. Kingsley, P.D., et al. Ontogeny of erythroid gene expression. *Blood* 121, e5–e13 (2013).
10. An, X., et al. Global transcriptome analyses of human and murine terminal erythroid differentiation. *Blood* **123,** 3466–3477 (2014).
